# The Role of Nerve Growth Factor (NGF) and Its Precursor Forms in Oral Wound Healing

**DOI:** 10.3390/ijms18020386

**Published:** 2017-02-11

**Authors:** Karl Schenck, Olav Schreurs, Katsuhiko Hayashi, Kristen Helgeland

**Affiliations:** 1 Department of Oral Biology, Dental Faculty, University of Oslo, PB 1052 Blindern, N-0316 Oslo, Norway; o.j.f.schreurs@odont.uio.no (O.S.); katsuh@jikei.ac.jp (Ka.H.); kristen.helgeland@odont.uio.no (Kr.H.); 2 Department of Dentistry, Jikei University School of Medicine 3-25-8, Nishi-Shimbashi, Minato-ku, Tokyo 105-8461, Japan

**Keywords:** saliva, salivary gland, keratinocytes, oral cavity

## Abstract

Nerve growth factor (NGF) and its different precursor forms are secreted into human saliva by salivary glands and are also produced by an array of cells in the tissues of the oral cavity. The major forms of NGF in human saliva are forms of pro-nerve growth factor (pro-NGF) and not mature NGF. The NGF receptors tropomyosin-related kinase A (TrkA) and p75 neurotrophin receptor (p75^NTR^) are widely expressed on cells in the soft tissues of the human oral cavity, including keratinocytes, endothelial cells, fibroblasts and leukocytes, and in ductal and acinar cells of all types of salivary glands. In vitro models show that NGF can contribute at most stages in the oral wound healing process: restitution, cell survival, apoptosis, cellular proliferation, inflammation, angiogenesis and tissue remodeling. NGF may therefore take part in the effective wound healing in the oral cavity that occurs with little scarring. As pro-NGF forms appear to be the major form of NGF in human saliva, efforts should be made to study its function, specifically in the process of wound healing. In addition, animal and clinical studies should be initiated to examine if topical application of pro-NGF or NGF can be a therapy for chronic oral ulcerations and wounds.

## 1. Introduction

Nerve growth factor beta (NGF) has been shown to play several important roles in wound healing in the skin. NGF is also widely expressed in the oral cavity as it is produced by oral keratinocytes and by stromal and infiltrating cells in the mucosal lamina propria, as well as being secreted into saliva. In contrast to the skin, however, little is known about the role of NGF in oral wound healing. This review summarizes knowledge about NGF in the oral cavity, describes its potential role in oral wound healing, and points to suggestions for further research into this area.

## 2. Sources of Nerve Growth Factor (NGF) in the Oral Cavity and the Salivary Glands

Early observations on NGF were derived from studies on snake venom and the mouse submaxillary gland [1]. In mice, NGF is synthesized as a large precursor molecule which is processed by alpha and gamma NGF subunits. However, the lack of alpha and gamma subunits in most other tissues and species, and the occurrence of many kallikreins expressed in the murine submaxillary gland that can convert the precursor, stand in contrast to what is seen in humans. This challenges the relevance of murine NGF as a proper model for studies that are relevant for the human situation [2]. In addition, later research has shown that precursor pro-nerve growth factor (pro-NGF) also displays biological activities and, in fact, is the main form produced in most tissues, including the brain [3].

**Figure 1:**
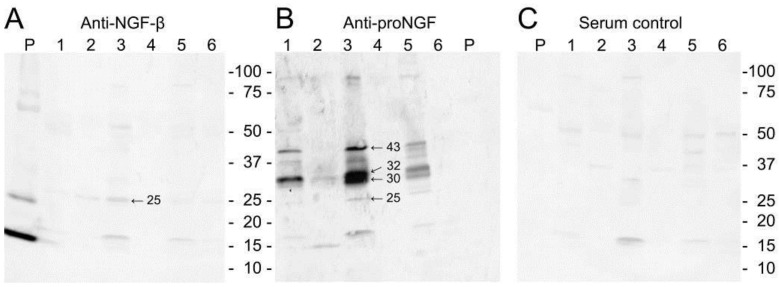
Western blotting of unstimulated whole saliva taken from six volunteers (lanes 1–6) and recombinant human nerve growth factor beta (NGF) (P) with antibodies reactive with (**A**) mature NGF or (**B**) pro-nerve growth factor (pro-NGF); (**C**) Control blot where primary antibody was substituted with normal rabbit serum. With permission from ref. [7].

NGF can be detected in human saliva [4,5,6,7] and, using enzyme-linked immunosorbent assay ELISA, human salivary NGF levels have been estimated to lie at approximately 1–10 ng/mL [5,6]. The main NGF forms in human saliva are, however, different types of pro-NGF and not mature NGF (Figure 1) [7], in contrast to what is seen in mice where the processed molecule is secreted [8]. The estimated molecular masses of 25, 32–34, and 43 kDa for glycosylated and unglycolysated pro-NGF forms (Figure 1) agree with molecular mass values previously described [8,9,10,11,12]. The ELISA kits used to determine NGF concentrations in saliva [5,6] are not capable of distinguishing between NGF and its pro-forms. In addition, measurement of NGF in saliva is subject to non-specific interference by constituents present in oral fluids [13]. Therefore, values of 1–10 ng/mL of mature NGF in human saliva, as estimated by ELISA, are likely to be too high.

Table 1 summarizes the sources of NGF and expression of its receptors in human salivary glands and the oral cavity.

**Figure 2:**
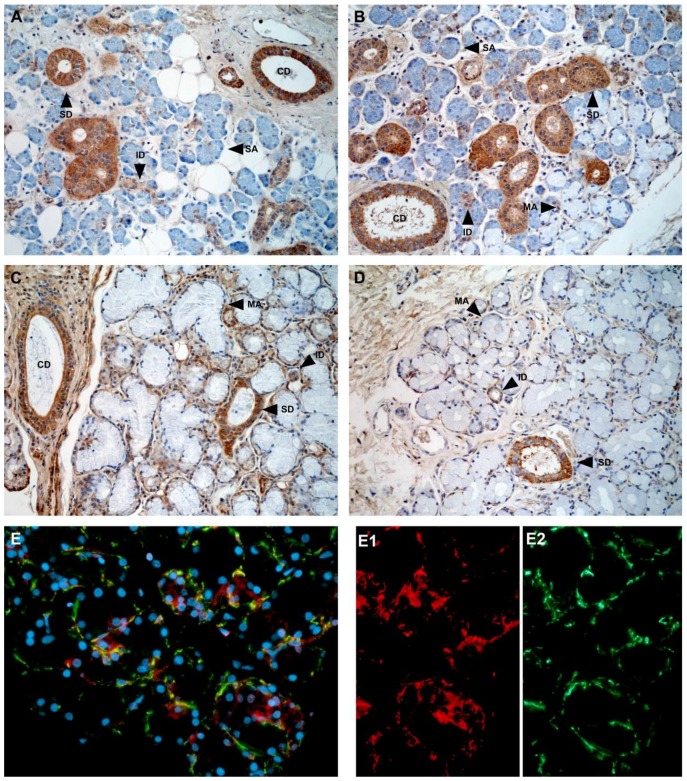
Immunohistological expression of pro-nerve growth factor (pro-NGF) in salivary glands. (**A**) parotis; (**B**,**E**) submandibular gland; (**C**) sublingual gland; (**D**) labial glands. CD, collecting duct; ID, intercalated duct; MA, mucous acinus; SA, serous acinus; SD, striated duct. The brown color indicates positive staining. Counterstaining with hematoxylin (blue), except for (**E**) where blue, red, and green colors represent nuclei, pro-NGF, and actin, respectively. Original magnification, 20 ×. With permission from ref. [7].

**Figure 3:**
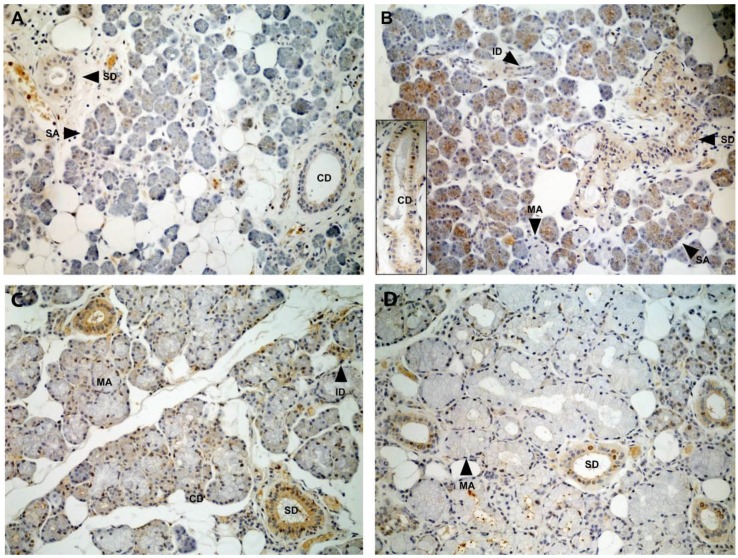
Immunohistological expression of nerve growth factor (NGF) in salivary glands. (**A**) parotis. (**B**) submandibular gland; (**C**) sublingual gland; (**D**) labial glands. CD, collecting duct; ID, intercalated duct; MA, mucous acinus; SA, serous acinus; SD, striated duct. The brown color indicates positive staining. Counterstaining with hematoxylin (blue). Original magnification, 20 ×. With permission from ref. [7].

The main form of NGF detected by immunostaining of biopsies from human salivary glands is pro-NGF and not mature NGF [7], in agreement with the finding that pro-NGF is the predominant NGF form in saliva. The main sites of pro-NGF production in the human salivary glands are the ductal components of the glands and not the acini: intercalated, striated, and collecting ducts in all human gland types (parotid, submandibular, sublingual and labial) show strong pro-NGF expression (Figure 2), but only weak cytoplasmic or sparse mature NGF (Figure 3) [7].

**Figure 4:**
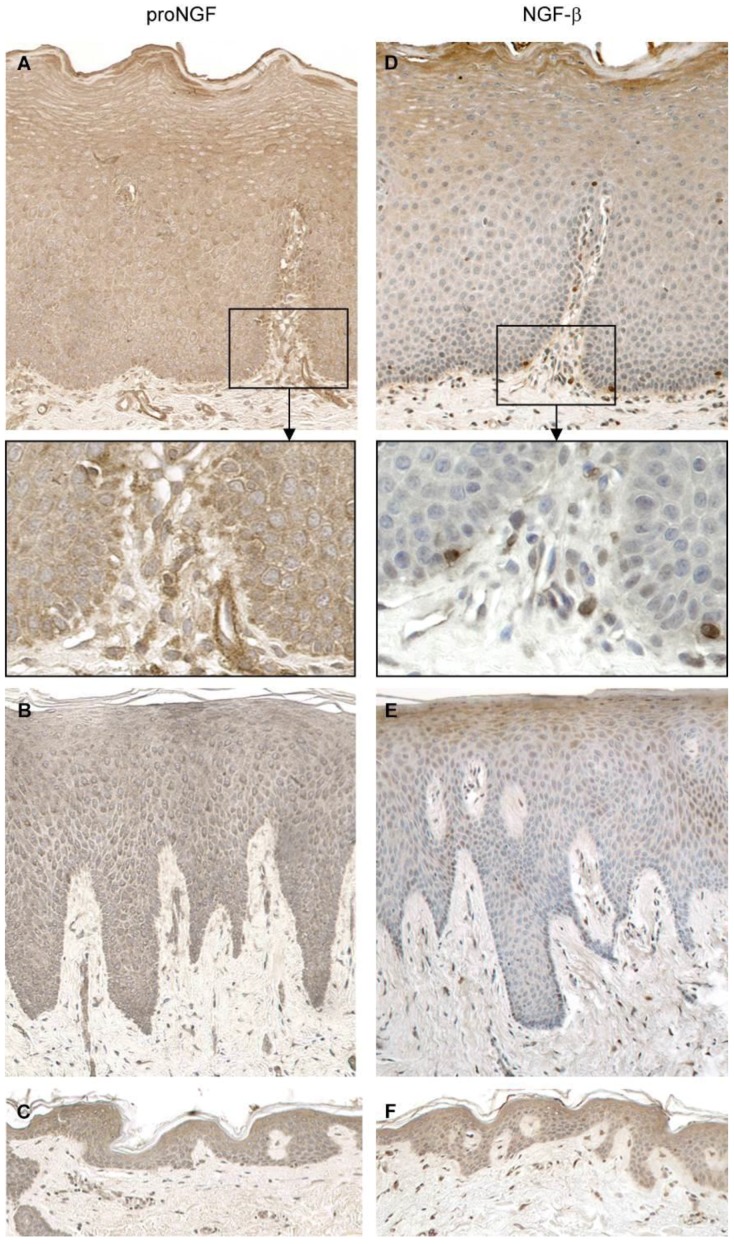
Immunohistological staining for pro-nerve growth factor (pro-NGF) (**A**–**C**) and NGF (**D**–**F**) in the epithelium of normal oral mucosa (**A**,**D**), gingiva (**B**,**E**), and skin (**C**,**F**). Original magnification, 20 ×. With permission from ref. [14].

The squamous epithelia of the oral cavity also produce forms of NGF. Immunostaining of biopsies from normal oral mucosa show the presence of pro-NGF in all epithelial layers, while mature NGF staining was observed in the granular and upper spinous cell layers (Figure 4) [14]. Leukocytes and fibroblasts, both in healthy and inflamed oral mucosa, can express both precursor and mature NGF forms [14,15].

## 3. Expression of NGF Receptors in the Oral Cavity and the Salivary Glands

**Figure 5:**
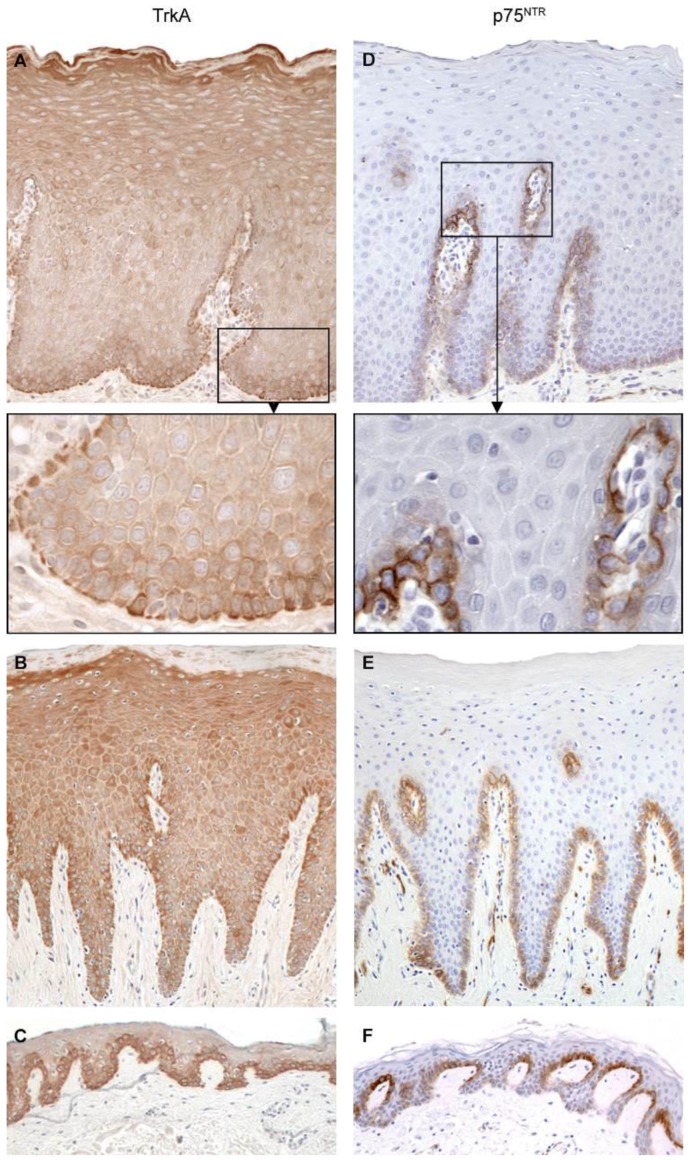
Immunohistological staining for pro-nerve growth factor NGF receptor tropomyosin-related kinase A (TrkA) (**A**–**C**) and NGF receptor p75 neurotrophin receptor (p75^NTR^) (**D**–**F**) in the normal epithelium from normal oral mucosa (NOM; **A**,**D**), gingiva (**B**,**E**), and skin (**C**,**F**). Original magnification, 20 ×. With permission from ref. [14].

NGF receptors are expressed throughout the soft tissues of the oral cavity. TrkA is expressed in basal and parabasal mucosal and gingival epithelial cells, with weaker expression in spinous and granular cell layers, while p75 neurotrophin receptor (p75^NTR^) only occurs in basal cell layers (Figure 5) [14]. p75^NTR^ is also expressed in oral squamous cell carcinomas (OSCCs) [16,17] and can be used as a prognostic marker together with the pattern of invasion. We found that high expression of p75^NTR^ in the central, superficial and invasive parts of the tumor was a marker for recurrence. The risk of recurrence was 6.4 times higher in patients expressing p75^NTR^ in more than 10% of tumor cells at the invasive front, compared with patients that had <10% positive tumor cells at this location [16].

**Figure 6:**
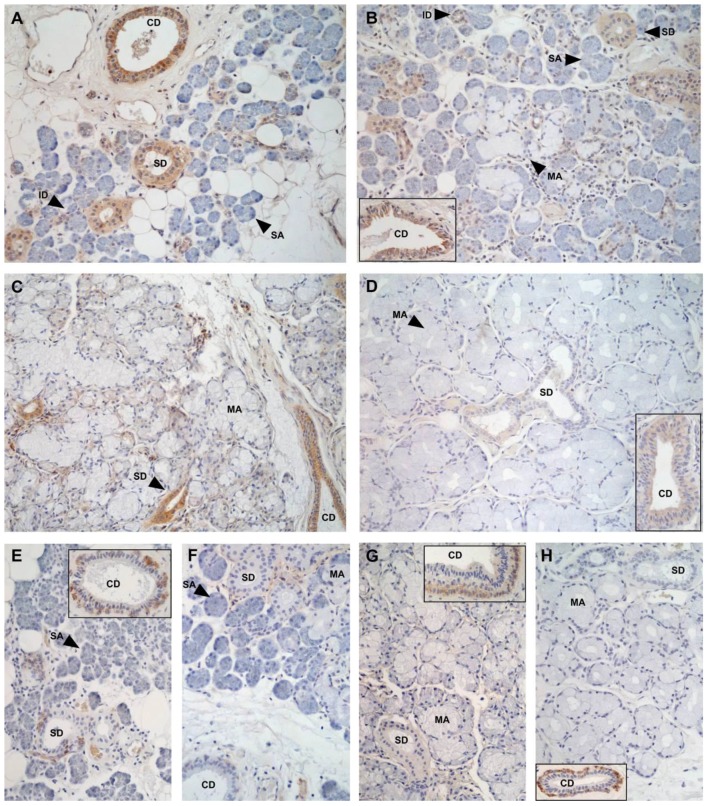
Immunohistological expression of TrkA (**A**–**D**), and p75^NTR^ (**E**–**H**) in salivary glands; (**A**,**E**) parotis; (**B**,**F**) submandibular gland; (**C**,**G**) sublingual gland; (**D**,**H**) labial glands. CD, collecting duct; ID, intercalated duct; MA, mucous acinus; SA, serous acinus; SD, striated duct. The brown color indicates positive staining. Counterstaining with hematoxylin (blue). Original magnification, 20 ×. With permission from ref. [7].

In human salivary glands, TrkA is strongly expressed in all ducts of all gland types, whereas p75^NTR^ expression is mainly confined to collecting ducts (Figure 6) [7]. Only sparse expression was seen in acini.

## 4. The Role of NGF in Different Processes Taking Place during Wound Healing in Squamous Epithelia

With regard to oral wound healing, the different forms of NGF present in saliva may act as “*luminal surveillance peptides*” that can reach their receptors only after exposure of the basolateral membrane due to local mucosal damage [18]. When oral epithelial layers are intact, epithelial cells and their tight junctions form a barrier that segregates salivary growth factors from their receptors. When the oral mucosa is wounded, growth factors in the oral cavity can reach their receptors that previously were hidden. This is suggested to occur in the gut: epidermal growth factor receptor is only expressed at the basolateral side of the epithelial cells and after wounding, epidermal growth factor (EGF) in the gut lumen can reach epidermal growth factor receptor, and help in the process of wound healing [18]. A similar scenario is likely to occur in the oral cavity. The mucosa is coated with saliva, and in case of wounding, pro-NGF and NGF in saliva can access NGF receptors on keratinocytes that are normally sealed off from the oral fluid compartment. Plasmin is generated due to local bleeding and this enzyme can cleave pro-NGF that is produced locally by the keratinocytes into mature NGF [11]. NGF can access TrkA on the basal keratinocytes of the wound edges and thereby enhance restitution and regeneration, as described below. Incidentally, this process is likely to be accompanied by a similar action of EGF. In the squamous oral epithelium of the oral mucosa, EGF receptors are also expressed in the lower epithelial layers [19] and can be exposed to salivary EGF [20] in case of wounding.

As discussed above, most of the NGF forms released in saliva are different NGF precursors that can be converted further by enzymes released at the site of activity. In general, the role of NGF in wound healing has mostly been studied using recombinant human mature NGF (rhNGF) and, as yet, no information is available with regard to the role of the NGF precursors in wound healing. Below, we review the action of rhNGF in in vitro models that mimic different stages or mechanisms of oral and skin wound healing, including restitution, cell survival, cellular proliferation, inflammation and tissue remodeling. A summary of the functions that NGF can have in oral wound healing is given in Table 2.

### 4.1. Restitution

**Figure 7:**
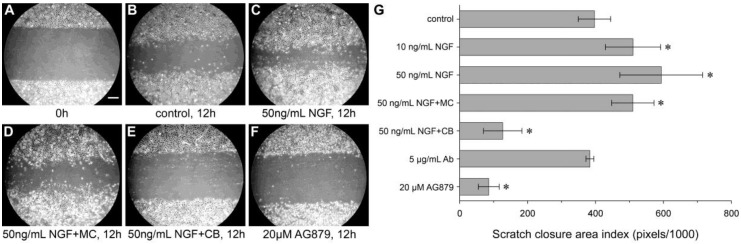
Effect of NGF and NGF-neutralizing agents on the migration of human oral mucosal keratinocytes. A scratch was made in confluent cultures of human oral mucosal keratinocyte monolayers. (**A**) Scratch at 0 h, scale bar 200 µm for **A**–**F**; (**B**–**F**) Migration after 12 h; (**B**) No addition; (**C**) Stimulation with 50 ng/mL of recombinant human NGF. Stimulation with 50 ng/mL of NGF and treatment with 5 µg/mL of mitomycin C (MC) (**D**) or 5 µM cytochalasin B (CB) (**E**) for 4 h before scratching and during further incubation with NGF; (**F**) Treatment with 20 µM TrkA inhibitor AG879; (**G**) Quantitative evaluation of scratch closure in three independent experiments performed in triplicate. Data are given as the mean ± standard deviation. * *p* < 0.05, as compared with the control. Ab, antibody. With permission from ref. [14].

A very early phase in epithelial wound healing, starting only minutes after wounding, is *restitution*, the rapid response from epithelial cells to migrate and cover the denuded or disrupted basal membrane. Thereby, fluid loss and infection are prevented (reviewed in [21]). NGF promotes cellular spreading, the process of releasing cells from their neighbors and allowing migration: after wounding, NGF up-regulates the expression of matrix metalloproteinase 9 (MMP-9; type IV collagenase), which cleaves important intercellular connections (α_6_β_4_ integrin in hemi-desmosomes and desmoglein in desmosomes, breaking down adherens junctions, desmosomes, and tight junctions) [22]. Furthermore, it has been documented that NGF increases the motility of a range of cell types, including human normal dermal keratinocytes [23], fibroblasts [24,25] and endothelial cells [26]. In an in vitro wound model (scratch closure), we showed that this also was the case for oral mucosal keratinocytes: a statistically significant greater scratch closure was seen after the application of recombinant human NGF (Figure 7) [14]. Together with other motogenic factors in saliva such as Trefoil Factor Family 3 (TFF3) [27], NGF therefore can be a factor in the rapid epithelial restitution seen in the oral cavity.

To complete restitution, the monolayer of flattened cells has to re-establish tight junction structure and cell polarity. In human mucosal epithelial cells from sino-nasal tissue, NGF up-regulates E-cadherin and zona occludens-1 expression strongly [28], indicating that NGF may play a role in re-establishing the mucosal epithelial barrier function after restitution.

### 4.2. Cell Survival

It is important that epithelial cells survive during restitution. In vitro studies show that autocrine NGF can act as a survival factor for human dermal keratinocytes through TrkA, possibly by preserving sufficient levels of the anti-apoptotic protein Bcl-2 [29]. We showed that oral epithelial cells also have the capacity to up-regulate anti-apoptotic proteins such as Inhibitors of Apoptosis Protein (IAP) and FLICE-Like Inhibitory Protein long form (FLIP_L_) proteins [30], but a connection with NGF and expression of anti-apoptotic proteins in oral keratinocytes has not been established.

### 4.3. Cellular Proliferation

After restitution, proliferation of different cell types, including keratinocytes, endothelial cells and fibroblasts, is essential for wound healing. NGF stimulates the proliferation of human normal skin keratinocytes in culture in a dose-dependent manner [23], and we found this also to be the case for human oral keratinocytes [14]. Regarding fibroblasts, the addition of exogenous NGF failed to stimulate dermal fibroblast proliferation. This was, however, probably due to sufficient endogenous production of NGF for normal cell proliferation, because blocking of the signaling pathway through inhibition of Trk or p75^NTR^ receptors reduced fibroblast proliferation, indicating that NGF also is a mitogenic factor for fibroblasts [25]. This agrees with the finding that exogenous NGF is mitogenic for fibroblasts isolated from the periodontal ligament [31]. Regarding endothelial cells, NGF is mitogenic [32], angiogenic [33] and suppresses endothelial apoptosis, probably via Vascular Endothelial Growth Factor A (VEGF-A) [34]. The proliferative capacity of NGF on endothelial cells from the oral cavity has not yet been examined, but it can be assumed that NGF also plays a role in angiogenesis after oral wounding.

### 4.4. Inflammation

Another early and important event in the epithelial wound healing process is the establishment and regulation of the activity of inflammation. Inflammation defends the host against infection, but it also has the function of resorbing and clearing away damaged cells and tissue components. Leukocytes including mast cells, eosinophils, macrophages and lymphocytes can produce NGF, while mast cells, eosinophils, basophils, neutrophils, macrophages and lymphocytes can express NGF receptors and, therefore, are targets for regulation by the peptide (reviewed in [35,36]). Pro-inflammatory cytokines such as Interleukin 1β (IL-1β) and Tumor Necrosis Factor α (TNF-α), which are released by several cell types after tissue damage, promote NGF synthesis [35]. NGF sustains the inflammatory process at several stages. It increases the expression of adhesion molecules such as Intercellular Adhesion Molecule 1 (ICAM-1) on endothelial cells and thus facilitates the egress of leukocytes from the blood stream into the damaged tissue [32]. It induces the release of inflammatory mediators from basophils [37] and mast cells [38], important cells with the capacity to detect tissue damage. NGF is chemotactic for and enhances superoxide production of neutrophils [35]. In oral mucosa, inflammatory cells in the epithelium and the connective tissue express NGF and its receptors, both in healthy and inflamed conditions [14,15]. Therefore, the above-mentioned responses are likely to occur in wounds of the oral cavity as well.

At the tissue level, inflammation induces the formation of granulation tissue. Neutrophils and mast cells are important mediator cells in this process at the early and late stages, respectively, and both cell types can be regulated by NGF as mentioned above.

### 4.5. Tissue Remodeling

The final stage in wound healing is the deposition of collagen and tissue remodeling. Fibroblasts have a central role in these processes. They synthetize and secrete collagens, proteoglycans, fibronectin and MMPs. NGF up-regulates the expression of MMP-9 [22]. During wound healing, different factors, including Transforming Growth Factor β1 (TGF-β1), promote fibroblast differentiation into proto-myofibroblasts and then myofibroblasts [39]. Myofibroblasts are key effectors in repair processes, as they control the deposition of components of the extracellular matrix, tissue contraction and wound resolution [39]. Their subsequent apoptosis is essential for adequate tissue re-epithelization [39]. Fibroblasts and myofibroblasts both express TrkA and p75^NTR^ receptors, but at markedly different levels: fibroblasts show the highest expression of TrkA, while p75^NTR^ is almost exclusively expressed in myofibroblasts [25]. NGF induces the differentiation of fibroblasts into myofibroblasts, similar to TGF-β1, and it stimulates dermal fibroblast contraction [25]. p75^NTR^ activation of myofibroblasts can lead to cell death and be one of the pathways that leads to elimination of the myofibroblasts, which is necessary for the completion of wound healing [25]. Wound healing in the oral cavity is rapid, and it occurs with little scarring. Regulation in oral wound healing by NGF of fibroblasts and myofibroblasts remains to be investigated.

## 5. Therapeutical Use of NGF in Chronic Ulcerations and Wounds

In the late 1970s, it was shown that when submandibular glands were removed in mice, the rate of contraction of experimentally induced wounds was retarded [40]. When NGF was topically applied, this accelerated wound healing in the sialoadenectomized animals [41], suggesting that NGF promotes cutaneous healing.

About twenty years later, similar results were obtained in humans. Research groups tested the application of murine NGF, isolated from the murine submaxillary gland (mNGF), as a treatment for human chronic corneal neurotrophic and chronic skin ulcers (reviewed in [42,43]). Both diseases are often unresponsive to conventional treatment and striking success was obtained by topical application of mNGF. After the finding that application of NGF into wounds in healing-impaired diabetic mice accelerated the rate of wound healing [44], the use of mNGF was also tested for treatment of corneal ulcers and of leg and foot ulcers in diabetic patients, again with good results (reviewed in [43]). Importantly, no systemic adverse events occurred during or after the treatment and no circulating antibodies against murine NGF were detected in patients treated with ocular topical application [43].

In the oral cavity, no studies have so far been carried out to examine whether NGF could be used in chronic mucosal ulcerations or wounds, such as oral mucositis, recurrent oral aphthous ulcers and bisphosphonate-associated osteonecrosis of the jaw. Oral mucositis is a severe side effect of radiotherapy and chemotherapy for cancer [45]. Cytokines and growth factors have been assessed for the management of oral mucositis in cancer patients and treatment with human recombinant keratinocyte growth factor (Palifermin) shows promising results [46]. Recurrent oral aphthous ulcers or recurrent aphthous stomatitis (RAS) are common conditions which have a significant impact on the patients’ quality of life, causing much pain and interference with mastication and speech [47]. Milder cases are treated with analgesia, corticosteroids and antimicrobial preparations. Additional topical therapies include other immunomodulators with anti-inflammatory effects such as calcineurin inhibitors, retinoids and tetracycline. There has also been increasing interest in the use of thalidomide for severe, recalcitrant RAS [47]. Bisphosphonates are the most common drugs for treatment of osteoporosis. Patients using the medication may show impaired wound healing following oral or periodontal surgery or endodontic therapy, with osteonecrosis of the jaw as a dreaded complication [48]. Given the good results obtained by the application of NGF on skin conditions, topical application of NGF could be tried out as a treatment for oral mucositis, recurrent oral aphthous ulcers and bisphosphonate-associated osteonecrosis of the jaw.

## 6. Future Directions

As pro-NGF appears to be the major form of NGF in the oral cavity, efforts should be made to study its function, specifically in the process of wound healing. Animal and clinical studies should be initiated to examine if topical application of high concentrations of pro-NGF or NGF can be a therapy for chronic oral ulcerations and wounds.

## Figures and Tables

**Table 1:** Sources of Nerve Growth Factor (NGF) and expression of its receptors in human salivary glands and the oral cavity.

Cell Type	Source of NGF/pro-NGF	NGF Receptor Expression
Salivary gland acini	±	±
Salivary gland ducts	+	+
Oral keratinocytes	+	+
Endothelial cells	+	+
Fibroblasts	+	+
Residential leukocytes	+	+
Infiltrating leukocytes	+	+

+ Strong expression; ± weak expression.

**Table 2:** The role of Nerve Growth Factor (NGF) in different processes taking place during wound healing in squamous epithelia (skin and oral mucosa).

Process in Wound Healing	Effects of NGF
Restitution	Cellular spreading, motility, re-establishment of epithelial barrier function
Cell survival	Up-regulation of anti-apoptotic proteins
Cellular proliferation	Proliferation of keratinocytes, endothelial cells and fibroblasts
Inflammation	Expression of adhesion molecules on endothelial cells, release of inflammatory mediators from basophils and mast cells, chemotaxis of neutrophils, formation of granulation tissue, angiogenesis
Tissue remodeling	Up-regulation of expression of matrix metalloproteinases, differentiation of fibroblasts into myofibroblasts, fibroblast contraction, apoptosis of myofibroblasts
